# Supplementary material for: Cilostazol effectiveness in reducing drug-coated stent restenosis in the superficial femoral artery: The ZERO study
Source: PLoS One. 2022 Jul 7;17(7):e0270992. doi: 10.1371/journal.pone.0270992 (PMC9262206; doi:10.1371/journal.pone.0270992)
Supplement: S1 File — (DOC) [file pone.0270992.s002.doc]

大腿膝窩動脈領域病変におけるシロスタゾール併用下薬剤溶出性ステント(Zilver PTX)の血管内治療の検討

研究実施計画書

主任研究者：

長野市民病院　循環器内科

三浦　崇

研究顧問：

長野赤十字病院　循環器内科

宮下　祐介

作成日

2018年4月23日　計画書案　第2版作成

秘密保持に関する供述：

本研究実施計画書は、本研究に直接係わる者および倫理審査委員会以外の者に情報を開示してはならない。また、本情報は事前の書面による主任研究者の承諾なしに本研究の実施あるいは評価以外の目的に利用してはならない。

遵守すべき諸規則：

本研究に関与するすべての者は「世界医師会ヘルシンキ宣言」および「臨床研究に関する倫理指針」に従う。

○目的

大腿膝窩動脈領域病変における薬剤溶出性ステントに対するシロスタゾールの再狭窄予防効果を検討する

○主な適格基準

- 大腿膝窩動脈領域にRutherford分類2～4の病変を有する慢性閉塞性動脈硬化症患者。ただし急性（7日以内）・亜急性（1ヶ月以内）下肢虚血症例は除外する。
- 年齢：同意取得時に20歳以上であること。性別：不問
- 12ヶ月以上観察が可能な患者（12ヶ月後のFollow　upが施行可能な患者）

○目標症例数

90例　：　**Zilver-PTX＋シロスタゾール群　90例**

○登録期間

2018年4月～2019年3月末

○研究期間

2018年4月～2020年3月末

○研究デザイン

多施設共同前向き試験

○研究スケジュール


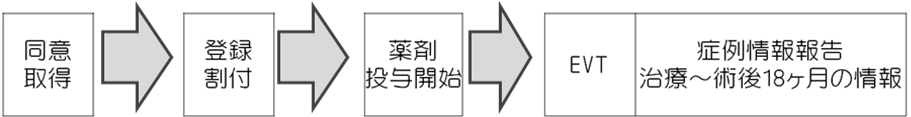


**症例情報報告**

**治療～術後12か月の情報**

**EVT**

○研究評価項目

主要エンドポイント

1. Primary Patency（PSVR≦2.0）：[標的病変部再狭窄](http://ejje.weblio.jp/content/標的病変部血行再建)からの回避

副次エンドポイント

1. 出血合併症(脳出血、消化管出血など、)
2. TVR（Target　Vessel　Revascularization）：標的血管の再血行再建術
3. MALE (Major Adverse Limb Event)：下肢関連死＋下肢大切断＋TLR＋出血合併症

1. ステント破損率
2. 心血管イベント
3. 有害事象

○連絡先

研究に関する問い合わせ

研究事務局

信州大学医学部　血管内治療学講座内

担当者：加藤　太門 栗原　真樹

〒390-0802 長野県松本市旭３丁目１−１

TEL：0263（37）3486（代表）　FAX：0263（37）3489

目次

1. 目的
2. 背景と根拠
3. 本研究で用いる規準・定義

3－1.心血管イベント

3－2.下肢血管イベント

3－3.有害事象

3－4.全死亡

3－5.ステント留置後再狭窄

3－6.ステント留置後再治療

1. 適格基準

4－1.選択基準

4－2.除外基準

1. 調査方法と研究スケジュール

5－1.患者同意

5－2.登録方法

5－3.研究デザイン

5－4.研究薬剤および使用方法

5－5.血行再建術

5－6.調査・検査項目スケジュール

1. 調査・検査項目および実施方法

6－1.実施時期の規定

6－2.患者背景

6－3.下肢血管内治療に関する調査項目

6－4.研究薬の投与状況

6－5.虚血症状

6－6.Ankle Brachial Pressure Index （ABPI）

6－7.ステント破損

6－8.臨床検査

6－9.心血管イベント

6－10.下肢血管イベント

6－11.有害事象

6－12.予期される有害事象

1. 中止
2. 目標症例数と研究機関

8－1.目標症例数

8－2.研究期間

1. エンドポイントの定義および安全性評価項目

9－1.主要エンドポイント

9－2.副次エンドポイント

9－3.安全性評価項目

1. 統計学的考察

10－1.目標例数の設定根拠

10－2.解析対象集団

1. 症例報告書

11－1.様式

11－2.入力方法（もしくは記載）

11－3.症例報告書の入力（もしくは記載）内容確認と問い合わせ

1. 倫理的事項

12－1.遵守すべき諸規則

12－2.説明文書・同意書（様式）の作成と改訂

12－3.個人情報の保護

1. プロトコールの承認と改訂

13－1.プロトコールの承認

13－2.プロトコールの改訂

1. 研究の終了と早期中止

14－1.研究の終了

14－2.研究の早期中止

1. 記録の保存
2. 研究費用の負担

16－1.資金源および起こりうる利害の衝突

16－2.治療に関する費用および健康被害に対する補償

1. 研究組織
2. 結果の発表
3. 文献
4. 奥付

付録1～7

略語一覧

EVT　　　Endovascular　　Therapy　　　　　　　　　　　　　血管内治療

PTA　　　Percutaneous　　transluminal　angioplasty　　経皮的血管形成術

TIA　　　Transient　ischemic　attack　　　　　　　　　　　 一過性脳虚血発作

ACD　　　Absolute　　claudication　　distance　　　　 絶対跛行距離

PSV　　　Peak Systolic Velocity　　　　　　　　　　 　 収縮期最大血流速度

1. 目的

大腿膝窩動脈領域病変における薬剤溶出性ステントに対してのシロスタゾールの再狭窄予防効果を比較する

こと。

1. 背景と根拠

近年、動脈硬化症疾患の増加に伴い下肢閉塞性動脈硬化症の増加が著しい。下肢閉塞性動脈硬化症は全身の動脈硬化症血管病変の一部分症であり重要臓器の虚血性合併症を認めることが多く、高齢者や全身状態が不良な例では、その治療としてより低侵襲の下肢血管内治療が選択されるところである。腸骨動脈領域においてはステントを用いた下肢血管内治療は確立したものとされている。しかしながら浅大腿動脈病変ではステント導入により、下肢血管治療の成績は飛躍的に向上したものの、長期開存率は十分とは言えない。その原因が線維芽細胞、平滑筋細胞を主体とする新生内膜過形成にあることは以前より指摘されており、2000年に発表されたTrans Atlantic　Inter-Society　Consensus（TASC）においても血行再建後の補助療法として、血管内膜過形成を阻害すると報告されている物質の臨床上の有効性を調べる必要性が懸案されている。

南都らは大腿膝窩動脈領域121病変のPTA施行例の後ろ向き解析においてシロスタゾールの再狭窄予防効果を報告しており、また同様の疾患について前向き試験において127症例を対象に3年まで経過観察した試験では、チクロピジン群よりシロスタゾール群において開存率が有意に高いことが報告された。さらに、EVT施行後1年がもっとも再狭窄の進行が高いとされているが、シロスタゾール群では特に最初の1年の再狭窄を抑えていることが明確になった。(1) また、飯田らは、大腿膝窩動脈領域に第１世代ナイチノールステントを留置した151例を対象にシロスタゾール群（シロスタゾール＋アスピリン）と非シロスタゾール群（アスピリン単剤）の再狭窄率の解析を行い、12ヶ月の再狭窄率は、シロスタゾール群21％、非シロスタゾール群48％であったと報告している。（2） さらには、宮下らはTCT（Transcatheter　Cardiovascular　Therapeuticｓ）2017において270例を対象に通常の抗血小板薬使用下に第２世代ナイチノールステント(Misagoステント)を使用した群、シロスタゾール併用下に第２世代ナイチノールステント(Misagoステント)を使用した群、アスピリン、プラビックス使用下に薬剤溶出性ステント(Zilver PTX)を使用した群の3群の１年間の再狭窄の解析を行い、29.1%、11.4%、21.3%であったことを報告している （DEBATE in SFA study）。　また、全らはZilver PTXの前向きレジストリー (ZEPHYR)の後ろ向き解析でZilver PTXのシロスゾールを投与した群としなかった群でプロペンシティマッチングを用いて背景を調整し１年の再狭窄を解析を行い、31%、 51%であったことを報告している。(3)

すなわち、シロスタゾールの第１、第２世代ナイチノールセルフステントに対する再狭窄予防効果は多くの研究で証明されている。　しかしながら、薬剤溶出性ステントに対するシロスタゾールの再狭窄予防効果は前向きには証明されていない。

以上より、シロスタゾール併用下に薬剤溶出性ステントを使用した群の単一群を前向き登録し、DEBATE in SFAのデータをヒストリカルデータとして、有効性、安全性を比較検討することにより、浅大腿動脈領域に対するさらなる至適血管内治療を検討する。

1. 本研究に用いる規準・定義

３－１．心血管イベント

TIAを含む虚血性脳卒中、出血性脳卒中、心筋梗塞、その他の血管事故のいずれかに該当するものとする（ここの診断基準については付録2．参照）

３－２．下肢血管イベント

アンプテーション（major／minor）、バイパス術への移行、再血行再建術（TLR*、TVR**、責任血管以外の血行再建術を含む）、ステント血栓症、下肢関連死、出血合併症のいずれかに該当するものとする。

* TLR（Target　Lesion　Revascularization）：標的病変の再血行再建術

**TVR（Target　Vessel　Revascularization）：標的血管の再血行再建術をいう。

TLR,TVRともにClinical drivenで実施することとする。

なお、同意取得時の両肢のEVT実施を予定している場合は、後のEVTをイベントとして取り扱わないこととする。

３－３．有害事象

研究薬剤の関連を問わず、研究期間中に発生した患者に不利益な全ての事象（臨床検査値の異常変動を含む）をいう。尚、本研究では「3－1．心血管イベント」は副次エンドポイント、「3－2．下肢血管イベント」は主要エンドポイントであるが有害事象としない。

３－４．全死亡

　死亡の理由を問わない全ての死亡とする。（なお、死亡原因による内訳は付録3参照とする）

３－５－１．ステント留置後再狭窄

　再狭窄：血管造影にて５０％以上の狭窄を確認したもの（Binary restenosis）、または下肢エコーにてPSVR>2.0のもの。

３－５－２．ステント留置血管の定義

　ステントを留置した部位の両側の健常部位から健常部位までの領域をいう

３－６．ステント留置術後再治療

　　血管内治療後に同血管に対し実施する治療目的の血管内治療、バイパス術をもって再治療と定義する。

1. 適格基準

４－１．選択基準

４－１－１．患者選択規準

次の全ての条件を満たす患者を研究対象とする。

1. 大腿膝窩動脈領域病変（Rutheford分類2～4）を有する慢性閉塞性動脈硬化症の患者。

ただし急性（7日以内）・亜急性（1ヶ月以内）の下肢虚血症例は除外する。

1. 年齢：20歳以上（同意取得時）
2. 性別：不問
3. 術後12ヶ月以上の観察が可能な患者（12ヶ月後のFollow upが可能な患者）

４－１－２．病変選択基準

1. 血管造影にて浅大腿動脈に有意な狭窄または閉塞病変でかつ新規病変に限る。なお、浅大腿動脈の下極は大腿骨上部の内転筋管の骨と重なる部分、上極は分岐部の起岐部とする。
2. 膝下動脈のrun-offが1本以上とし、flow limitingのない狭窄病変であれば可能とする。さらに、両側に病変を合併する患者、大動脈-腸骨動脈領域病変を合併する患者も対象とする。ただし、両側に病変を有する患者においての血管内治療は30日～45日の間隔をおいて各肢の治療を行うこととする。
3. 閉塞病変も対象とする。

４－２．除外基準

４－２－１．患者除外基準

次のいずれかの条件に該当する患者は研究対象外とする。

1. 出血している又は出血傾向のある患者

（血友病、毛細血管脆弱症、頭蓋内出血、消化管出血、尿路出血、喀血、硝子体出血等の出血）

1. 重症なうっ血性心不全の患者
2. 冠動脈へのDES（Drug Eluting Stent）埋め込み後で抗血小板薬を単剤減量できない患者
3. 抗凝固薬(ワーファリン、プラザキサ、イグザレルト)使用中の患者
4. 研究薬剤の成分に対し重篤な副作用、過敏症の既往歴がある患者
5. 妊娠又は妊娠している可能性のある患者
6. 急性・亜急性下肢虚血の患者
7. その他、担当医師の判断により、研究対象として不適切と考えられる患者

４－２－２．病変除外基準

次のいずれかの条件に該当する病変は研究対象外とする。

1. Inflow（大動脈―腸骨動脈病変）病変が残存しているもの。ただし、同時期治療を行えば研究対象としてもよいものとする。

2．　膝下動脈のrun-offが1本未満

1. 調査方法と研究スケジュール

５－１．患者の同意

　研究責任（担当）医師は、下記の内容について患者本人に説明し、参加について文書による同意を患者本人より得るものとする。

1. 研究の目的
2. 研究方法
3. 予想される臨床上の利益および危険性
4. 他の治療法の有無
5. 研究への参加に同意しない場合であっても不利益は受けないこと
6. 研究への参加をいつでも撤回出来ること
7. 中止の条件又は理由
8. 研究に参加した場合の費用負担は通常の保険診療範囲であること
9. 研究に伴う補償の有無
10. プライバシーは守られること。
11. 本研究は倫理委員会により承認されたものであること
12. 研究期間と参加予定人数
13. 研究のスケジュール
14. 問い合わせ等の連絡先

５－２．登録方法

　研究責任医師は研究を実施する前に各施設の倫理審査委員会に申請し承認を得る。

研究責任（担当）医師は、各施設の倫理審査委員会での承認が得られた後、倫理委員会通過証明書を登録センターにメールで送付する。

症例登録および研究薬剤の投与は、登録センターマニュアルに従うものとする。

登録センター 登録事務局

信州大学医学部　血管内治療学講座内

〒390-0802 長野県松本市旭３丁目１−１

TEL：0263（37）3486（代表）　FAX：0263（37）3489

担当者　栗原　真樹　(junkan@shinshu-u.ac.jp

５－３．研究デザイン


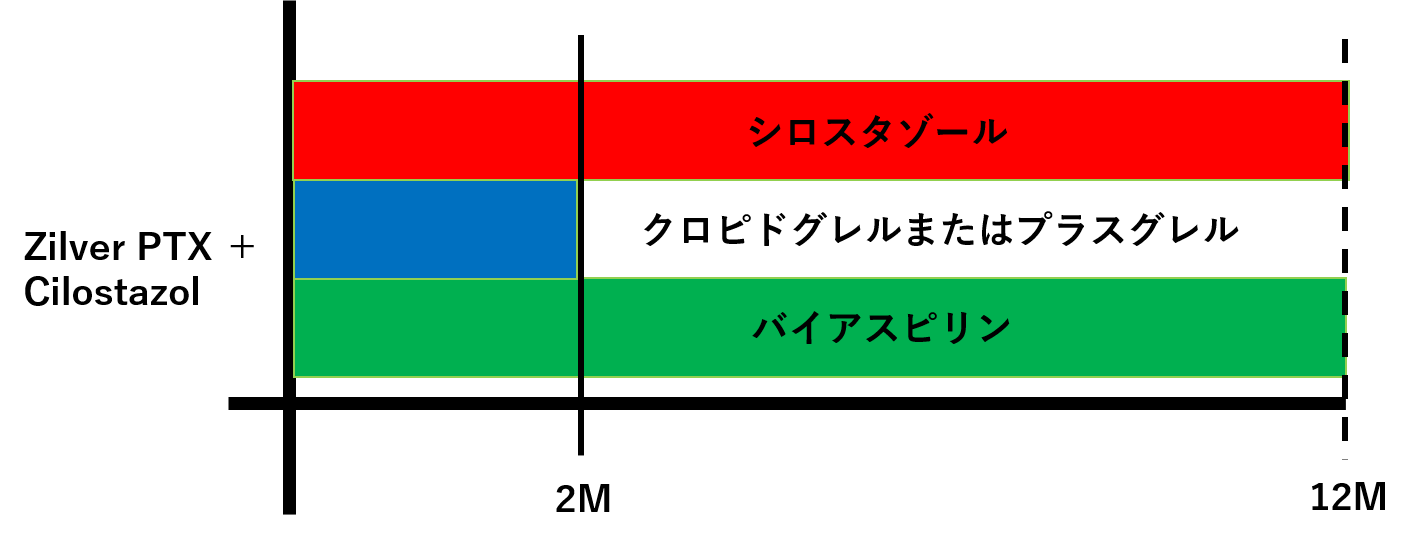


５－４．研究薬剤および使用方法

1. AAA

バイアスピリンは100mg 分1、クロピドグレルは75mg 分1、プラスグレルは 3.75mg分1、シロスタゾールは200mg 分2とする。　なおEVT実施の3～7日以上前より経口投与を開始する。 EVT２か月後クロピドグレルまたはエフィエントを中止する。

シロスタゾール投与開始後、頭痛等の副作用発現により減量をせざるを得なくなった場合には、1日100mg分2までの減量は可能とする。

1. 併用薬剤

原則として、以下のアスピリン・クロピドグレル・プラスグレル・シロスタゾール以外の抗血小板薬は研究期間中に使用しないこととする、ただし、やむを得ない場合には併用可とするが、その理由については医師コメント欄に記載する。　なお、塩酸チクロピジン（パナルジン）はクロピドグレルへ変更する。

５－５．血行再建術

血行再建術については、アプローチは問わない。

サイズは対象血管のreference径で4mm以上とする。なお、病変長は36cm未満までとする。ステント適応はバルーン拡張後の圧較差10mmHgまたは％DS＞30％、Flow Limiting dissectionとする。

今回の臨床研究において、同意取得時に両肢のEVT実施を予定している場合、両肢のEVT実施の期間は30～45日を超えないものとする。なお、本研究における観察肢は先にEVTを実施した肢を登録することとし、後に実施した肢のEVTはイベントとして取り扱わないこととする。

５－６．調査・検査項目スケジュール

〔観察項目および観察時期〕

|  |  | 定期検査（同意取得後） | | | | | |
| --- | --- | --- | --- | --- | --- | --- | --- |
| 項目／時期 | 事前検査時 | 術直後 | １M | ３M | ６M | １２M | 投与中止もしくはイベント発症時 |
| 患者背景 | ● |  |  |  |  |  |  |
| 虚血症状 | ● |  | ○ | ● | ● | ● | ● |
| 臨床検査値 | ○ |  | ○ | ○ | ● | ● | ● |
| イベント　※２ |  | | | | | | |
| 有害事象 |  | | | | | | |
| デバイス情報　※１ |  | ● |  |  |  |  | ● |
|  |  |  |  |  |  |  |  |

●；必須、○非必須（ただし臨床検査については出来る限り実施すること）

※１：ステント破損率の評価をX線にて実施

※２：イベントは心血管イベント、下肢血管イベント、全死亡。

†DES留置後、１か月以上経過した症例で、主治医が抗血小板薬単剤へ減量可能と判断した症例はエントリー可とする。

1. 調査・検査項目および実施方法

６－１．実施時期の規定

各調査の実施時期の定義は下記の通りとする。

| 項目 | 定義 |
| --- | --- |
| 術直後 | 下肢血管内治療終了後、治療室退出まで |
| 術後1ヶ月 | 術直後から1ヶ月±2週間 |
| 術後3ヶ月 | 術直後から3ヶ月±1ヶ月 |
| 術後6ヶ月 | 術直後から6ヶ月±1ヶ月 |
| 術後12ヶ月 | 術直後から12ヶ月±1ヶ月 |

６－２．患者背景

基本情報

- 被験者識別コード（「付録7．　匿名化番号対照表」参照）
- 治療年月日
- 治療時年齢
- 性別
- 併用薬剤名、投与量、開始日、終了日
- 身長、体重、年齢
- Rutherford分類

既往歴・合併症・喫煙率

- 高血圧　：　有、無
- 出血　：　有、無
- 糖尿病　：　有、無
- 脂質異常症　：　有、無
- 冠動脈疾患　：　有、無
- 脳卒中・TIA既往　：　有、無
- 喫煙歴　：　有、無
- 透析　：　有、無

既往歴・合併症の定義

| 既往歴・合併症 | 定義 |
| --- | --- |
| 高血圧 | 血管内治療実施前3ヶ月以内のいずれかの診察時に収縮期血圧140mmHg以上または拡張期血圧90mmHg以上または降圧を目的とした降圧薬の内服、これらのいずれかを満たすもの。 |
| 出血 | 入院を要する、または輸血を要するような出血の既往 |
| 糖尿病 | 血管内治療実施前3ヶ月以内のいずれかの診察時に空腹時血糖126mg/dL以上または随時血糖200mg/dL以上または血糖降下薬の内服、インスリン製剤の投与、以前に医療機関で糖尿病と診断された、これらのいずれかを満たすもの。 |
| 脂質異常症 | 血管内治療実施前3ヶ月以内のいずれかの診察時にLDLコレステロール≧140mg/dL、HDLコレステロール＜40mg/dL、トルグリセリド≧150mg/dL、または、脂質改善薬の内服、これらのいずれかを満たすもの。 |
| 冠動脈疾患 | 過去に急性心筋梗塞、狭心症、冠動脈血管形成術、冠動脈バイパス術を受けたことがあるもの。 |
| 脳卒中・TIA既往 | 過去に脳梗塞、脳出血、くも膜下出血あるいはTIAの治療を受けたことがあるもの。 |
| 喫煙歴 | 血管内治療前1年以内に平均1本/日以上の喫煙習慣を有するもの。 |

６－３．下肢血管内治療に関する調査項目

下肢血管内治療情報（デバイス情報を含む）

□　治療足（手技毎）

：　左、右

：　Type（TASC分類*　A、B、C、D）

*TASC分類はTASCⅡに基づく

：　血管経（mm）、長さ（mm）、狭窄率（％）　治療時には定規を貼ってください。

：　run　offの状態（本数１、２、３）

- 膝下動脈（前脛骨・後頸骨・腓骨動脈）の病変*部位

　*血流低下を合併する狭窄もしくは閉鎖

- デバイスの種類

ステント、バルーン、その他

- ステント経（mm）
- ステント長（mm）
- ステント本数（本）
- 前拡張　：　最大バルーン経（mm）
- 後拡張　：　最大バルーン経（mm）
- 手技に伴う合併症発症　：　有、無

＜ありの場合＞

□内容　：　出血、血腫、輸血、末梢塞栓、脂肪、下肢切断、緊急外科手術

　　□　末梢側への塞栓　：　有、無、発症日

　　□　出血性合併症　：　有、無（輸血、外科的修復を要する穿刺部の出血）、発症日

６－４．研究薬の投与状況

- シロスタゾール（開始日、終了日、１日用量）
- アスピリン（開始日、終了日、１日用量）
- 硫酸クロピドグレル（開始日、終了日、１日用量）

６－５．虚血症状

- 冷感　：　－、±、＋、＋＋、＋＋＋
- しびれ感　：　－、±、＋、＋＋、＋＋＋
- 間欠性跛行　：　Rutherford分類

で評価する。

６－６．Ankle　Brachial　Pressure　Index（ABPI）比

- ABPI

（日本脈管学会間歇性跛行重症度評価小委員会推奨）

６－７．ステントの破損

- ステントのX線写真によりストラットの破断の有無の確認を行い、破断の程度をタイプ0～Ⅳで判断する。（付録4参照）

６－８．血管エコー

病変部 PSV、病変部PSVR

６－９．臨床検査

1. 血液学的検査

赤血球数、白血球数、血小板、Hb、Ht

1. 生化学的検査

総蛋白、アルブミン、総ビリルビン、AST（GOT）、ALR（GPT）、Al-p、γ-GTP、BUN、クレアチニン、LDLコレステロール、中性脂肪、HDL-コレステロール、空腹時血糖、HbA1c、CRP

　　６－１０．心血管系イベントおよび全死亡

- 有、無
- イベント発症日／最終フォローアップ確認日

「あり」の場合は初回イベント発症日を、「なし」の場合は最終フォローアップ確認日を記載する。

≪　「あり」の場合　≫

- イベント内容

TIAを含む虚血性脳卒中、出血性脳卒中、心筋梗塞、その他の血管事故（大動脈解離または破裂、肺塞栓症、または四肢梗塞）および全死亡（脳卒中死、心血管、その他、不明）。尚、複数のイベントが発生した場合は最初のイベント発生日と確認されたイベントを記載する。

　　6-11．下肢血管イベント

- 有、無
- イベント発症日／最終フォローアップ確認日

「あり」の場合は初回イベント発症日を、「なし」の場合は最終フォローアップ確認日を記載する。

＜　「あり」の場合　＞

- イベント内容：アンプテーション（major／minor）、バイパス術への移行、再血行再建術（TLR*、TVR**、責任血管以外の血行再建術を含む）、ステント血栓症、下肢関連死、出血合併症

尚、複数のイベントが発症した場合は最初のイベント発生日と確認された全てのイベントを記載する。

6-12．有害事象

　　　研究期間中、研究薬投与前に診られなかった全ての異常所見（死亡を含む）および臨床検査値の異常変動が認められた場合とする。ただし心血管イベントおよび下肢血管イベントは含まない。

6-13．予期される有害事象

　　　研究薬に関連して予期される有害事象は付録6を参照

1. 中止

以下の場合はその症例の研究継続を中止する。

1. 患者の同意撤回
2. 患者が理由不明で来院しなくなった場合
3. ２）以外でも研究責任（担当）医師が継続困難と判断した場合
4. 研究全体の終了
5. 目標症例数と研究期間

８－１．目標症例数

90例

８－２．研究期間

2018年4月～2020年3月末　（症例登録期間は、2018年4月～2019年3月末とする。）

1. エンドポイントの定義および安全性評価項目

９－１．主要エンドポイント

　Primary Patency ：[標的病変部再狭窄](http://ejje.weblio.jp/content/標的病変部血行再建)からの回避　(血管超音波におけるPSVR≧2.0からの回避)

MALE (Major Adverse Limb Event)：下肢関連死＋下肢大切断＋TLR＋出血合併症

９－２．副次エンドポイント

1. 出血合併症(脳出血、消化管出血など、)
2. TVR（Target　Vessel　Revascularization）：標的血管の再血行再建術
3. MALE (Major Adverse Limb Event)：下肢関連死＋下肢大切断＋TLR＋出血合併症

④　ステント破損率

⑤ 心血管イベント

⑥ 有害事象

９－３．安全性評価項目

1. 薬剤の途中中断
2. 有害事象

10．　統計学的考察

　　１０－１．目標症例数の設定根拠

宮下らは、TCT（Transcatheter　Cardiovascular　Therapeuticｓ）２０１7において、大腿膝窩動脈領域に

270例を対象に通常の抗血小板薬使用下に第２世代ナイチノールステント(Misagoステント)を使用した群、

シロスタゾール併用下に第２世代ナイチノールステント(Misagoステント)を使用した群、アスピリン、プラビッ

クス使用下に薬剤溶出性ステント(Zilver PTX)を使用した群の3群の再狭窄の解析を行い、次のように報告

している。１２ヶ月の再狭窄率は、非シロスタゾール内服下第２世代ナイチノールステント群29.1%、シロスタ

ゾール内服下第２世代ナイチノールステント群11.4%、非シロスタゾール内服下薬剤溶出性ステント群

21.3%である。　この研究では１群 90症例を登録し解析を行った。このデータをヒストリカルデータとして使

用するため、本研究でも目標症例を90症例と設定する。

　１０－２．解析対象集団

　　　　本研究は、登録された患者のうち適格基準に合致する全患者を解析対象集団とする。

１１．　症例報告書

　　１１－１．様式

　　　症例報告書（CRF）は研究事務局から交付する書類にて、データを記載したものとする。またデータ入力後のプリントアウトしたものも含まれる。

　　１１－２．入力方法（もしくは記載）

　　　症例報告書に入力の際には以下の事項を遵守すること。

　　　・　入力と訂正は研究責任（担当）医師が行う。

- 入力時は「付録7．匿名化番号対照表」を参照しカルテが当該被験者のものであることを確認する。
- 症例報告書はデータ入力終了後、プリントアウトしカルテ等に保管する。
- 入力方法に関して質問がある場合は、研究事務局に問い合わせる。

　　１１－３．症例報告書の入力（もしくは記載）内容確認と問い合わせ

　　　　研究事務局はデータ入力後、プリントアウトしカルテ等に保管する。

- 入力の不備
- プロトコールの整合性
- 症例報告書の入力内容間の整合性

研究事務局は、照会すべき点をデータ照会票にまとめ、研究責任医師に送付する。

研究責任（医師）は、入力及び訂正を行うかデータ照会票に回答を記入し、研究事務局に返信の上、主任研究者より研究終了の連絡があるまで保管する。

12．　倫理的事項

　　１２－１．遵守すべき諸規則

　　　　本研究に関与する全ての者は「世界医師会ヘルシンキ宣言（2002年10月改訂）」および「臨床研究に関する倫理指針（文部科学省・厚生労働省、平成26年１２月22日（平成29年2月28日一部改訂）を遵守して研究を行う。

　　１２－２．説明文書・同意書（様式）の作成と改訂

　　　　説明文書・同意書（様式）および同意撤回書は研究責任（担当）医師が作成する。ただし、主任研究者が見本として作成した「説明文書・同意書（様式）を修正して用いてもよい。また作成した説明文書・同意書（様式）は研究開始前に所属する医療機関の倫理審査委員会に提出し、その承認を得る。

　　　　研究開始後に研究責任（担当）医師が被験者の同意に関連する新たな知見を得、説明文書・同意書（様式）の改訂が必要と判断した場合には、それを改訂する。被験者の同意に関連する新たな知見とは、例えば当該治療法に関連する新たな有害事象の情報、あるいは当該疾患に関わる新治療法等の開発に関する情報などを指す。なお、改訂の内容を重大と判断する場合は所属する医療機関の倫理審査委員会に提出し、その承認を得る。

　　１２－３．個人情報の保護

　　　　倫理審査委員会や研究に携わる関係者により原医療記録を閲覧出来る。

　　　　研究に携わる関係者は被験者の個人情報保護に最大限の努力をはらう。

　　　　研究責任（担当）医師は、症例登録票及び症例報告書等を当該医療機関外に提供する際には、連結可能匿名化を行う為に新たに被験者識別コードを付し、それを用いる。医療機関外の者が被験者を特定出来る情報（氏名・住所・電話番号など）は記載しない。

　　　　登録センターが医療機関へ照会する際の被験者の特定は、研究責任（担当）医師が管理する被験者識別

コードを用いて行う。

　　　　主任研究者等が研究で得られた情報を公表する際には被験者が特定できないよう十分に配慮する。

13．　プロトコールの承認と改訂

　　１３－１．プロトコールの承認

　　　　本研究においては、プロトコールが本研究参加施設の倫理審査委員会もしくはそれに準ずる組織の審査を受け、承認された後に本研究参加施設の施設登録が行われる。施設登録が完了した後、本研究参加施設で研究が実施される。

　　１３－２．プロトコールの改訂

　　　　本研究開始後、プロトコールの改訂が必要となった時、改訂の内容に応じて研究の中止、継続を決定し、その旨を研究事務局より各研究参加施設に連絡する。

　　　プロトコールの変更は主任研究者が招聘するプロトコール検討委員会（メンバー：主任研究者、副主任研究者、統計解析責任者、その他必要とする委員）で決定する。検討結果は長野市民病院倫理審査委員会に提出し承認を得るものとする。また本研究参加施設の倫理審査委員会もしくはそれに準ずる組織の承認を得た後に、新たなプロトコールのもと本研究参加施設で研究を実施・再開する。

14．　研究の終了と早期中止

　　１４－１．研究の終了

　　　　データベースの固定を持って研究の終了とし、主任研究者はその旨を本研究参加施設の責任医師および統計解析責任者に報告する。

　　１４－２．研究の早期中止

　　　　研究継続の是非を議論する必要が生じた場合には、主任研究員が効果および安全性評価委員、統計解析責任者と協議する。

15．　記録の保存

　　　　主任研究者、研究責任（担当）医師および研究事務局は、本研究の実施に係る記録（文書および電子記録）を研究終了後3年間保存する。

16．　研究費用の負担

　　１６－１．資金源および起こりうる利害の衝突

　　　　本研究は、信州大学医学部循環器内科学講座（内科学第5）の支援を受けて行われる。尚、本研究の結果にかかわらず、それが信州大学医学部循環器内科学講座（内科学第5）に何らかの利益や損害を受けることはない。

１６－２．治療に関わる費用および健康被害に対する補償

　　　　この研究は通常の健康保険の範囲内で行われ、研究にかかる観察・検査、使用薬剤等には被験者の健康保険が適用される。また、本研究で行われる治療は通常の診察で一般的に行われるものであり、通常の診察を上回る危険性はない。

17．　研究組織

　　　主任研究者

　　　　三浦　崇　　　長野市民病院　循環器内科

　　　　　　　　　　　　　〒381-0006 長野県長野市富竹1333-1

　　　　　　　　　　　　　TEL：0262（95）1199（代表）　FAX：0262（95）1148

　　　副主任研究者

朴澤　耕治 新東京病院　循環器内科

　　　　　　　　　　　　　〒270-2232　千葉県松戸市和名ヶ谷1271

　　　　　　　　　　　　　TEL：047-711-8700

加藤　太門　　　信州大学医学部附属病院　循環器内科

　　　　　　　　　　　　　〒390-8621 長野県松本市旭3−１−1

　　　　　　　　　　　　　TEL：0263（37）3486（代表）　FAX：0263（37）3489

　研究顧問

宮下　祐介　　信州大学医学部附属病院　循環器内科

　　　　　　　　　　　　　〒390-8621 長野県松本市旭3−１−1

　　　　　　　　　　　　　TEL：0263（37）3486（代表）　FAX：0263（37）3489

曽我　芳光　　小倉記念病院　循環器内科

　　　　　　　　　　　　　〒802-8555 福岡県北九州市小倉北区浅野3−2−1

　　　　　　　　　　　　　TEL：093（511）2000（代表）

研究補佐

栗原　真樹　　　信州大学医学部附属病院　循環器内科

　　　　　　　　　　　　　〒390-8621 長野県松本市旭3−１−1

　　　　　　　　　　　　　TEL：0263（37）3486（代表）　FAX：0263（37）3489

　　　効果および安全性評価委員

　　　　順天堂大学医学部附属医院　循環器内科　　　　　　　　　　　葛西　隆敏

18．　結果の発表

　　　プロトコール検討委員会は、研究結果の論文投稿および公表に責任を持って対応する。また研究参加者の秘密保持に責任をもつ。

19．　文献

１） Iida O, Nanto S et al.: J Vasc Surg. 2008; 48(1):144-9

2) Iida O, et al.: Circulation. 2013; 127: 2307-2315

3) Zen K, et al.: J Vasc Surg. 2017; 65: 720–5.

20．　奥付

　　　本研究実施計画書の版権は主任研究者に帰属し、その全部または一部を無断で転用することを禁ず。

　　　　版権所有者：

　三浦　崇　　　長野市民病院　循環器内科

　　　　　　　　　　　　　〒381-0006 長野県長野市富竹1333-1

　　　　　　　　　　　　　TEL：0262（95）1199（代表）　FAX：0262（95）1148

付録１．　Rutherford分類

[Rutherford RB et al: Suggested standards for reports dealing with lower extremity ischemia. J Vasc Surg 26:517,1997]

| 度 | 群 | 臨床定義 | 客観的基準 |
| --- | --- | --- | --- |
| 0 | 0 | 無症状　循環動態からみても有意な閉塞性病変なし | トレッドミル運動負荷試験あるいは反応性充血試験正常 |
|  | 1 | 軽度跛行 | トレッドミル運動負荷試験終了可(*)、運動後のAP＞50mmHg、　しかし安静時に比して最低20mmHg下降 |
| I | 2 | 中等度跛行 | 1群と3群の中間 |
|  | 3 | 高度跛行 | 標準的トレッドミル運動負荷試験終了不能(*)および運動後のAP＜50mmHg |
| II | 4 | 虚血性安静時痛 | 安静時AP＜40mmHg、足関節あるいは中足骨PVRの平坦化あるいは波高の激減 TP＜30mmHg |
| III | 5 | 軽度組織消失　非治癒性潰瘍、後半足虚血を伴う限局性壊疽 | 安静時AP＜60mmHg、　足関節あるいは中足骨PVRの平坦化あるいは波高の激減 TP＜40mmHg |
|  | 6 | 広範な組織喪失　TMよりも高位に拡大、もはや機能的足部リム・サルベージ不能 | 5群と同じ |

付録2.　心血管イベント診断基準

虚血性脳卒中

　神経学的機能障害をWholey et al.〔１０〕の報告に伴い、一過性脳虚血発作（TIA）、軽微な神経学的機能障害（７日以内に寛解もしくはNIH stroke scale４点未満の悪化が持続）をｍｉｎｏｒ ｓｔｒｏｋｅ、神経学的機能障害が７日後も持続し、NIH stroke scale ４点以上に悪化するものをｍａｊｏｒ ｓｔｒｏｋｅに分類する。

一過性脳虚血発作（ＴＩＡ）

　本研究ではＴＩＡの診断基準を画像所見の有無に関わらず“神経症状の持続が２４時間未満のもの（ＣＡＳ手技による術中の血行遮断によるものと思われるものを除く）”とする。神経症状を伴わない意識障害やけいれん、閃輝性暗点、単独のめまい・複視・構語障害などの症状をもってＴＩＡと診断してはならない。画像所見の有無は参考としない。

出血性脳卒中

　ＣＴまたはＭＲＩによって診断された症候性のクモ膜下出血および脳内出血。症状が頭痛のみで他の他覚的神経症状を伴わない場合は、これに該当しない。

心筋梗塞

　ＡＨＡ／ＡＣＣガイドラインに従い、以下に示す　１～４のいずれかをもって心筋梗塞と診断する。ただし、臨床的心筋炎、心膜炎と診断されたものは除外する。

1. 新たな異常Ｑ波の出現
2. 定型的または非定型的な胸部症状を有し、虚血性心電図変化があり、かつ心筋逸脱酵素の上昇《施設基準の２倍を超えるＣＫ（ＣＰＫ）またはＣＫ－ＭＢ（ＣＰＫ－ＭＢ）の上昇》を伴うもの
3. 定型的胸部症状および心筋逸脱酵素の上昇を伴うもの
4. 活字綱剖検所見（組織学的な新鮮梗塞または最近生じた冠動脈閉塞所見）

その他の血管事故

　大動脈解離または破裂、肺塞栓症、臓器または四肢梗塞とする。

付録３．　全死亡

脳卒中死　：　虚血性脳卒中、出血性脳卒中で死亡

心血管死　：　心筋梗塞とその他の血管事故における死亡

その他の死亡　：　その他の原因による全ての死亡

下肢関連死　： 下肢の血管事故、感染における死亡

　　　　不明　：　死亡の原因が明らかでないもの

付録４．　ステントの破損

　Ｘ線撮影にてステント全体のストラットが見えるように、解像度は可能な限り最高の解像度で行う。

また、下肢屈曲および進展時の両方のステントで判断し、ステント像の欠落や骨との重なりのない正面像およびその直角の２方向から撮影した画像を元に判断する。なお画像は高画質スクリーン（フィルム使用時）かそれと同等のヒストグラム（コンピュータＸ線撮影：ＣＲ）を使用することとする。さらに、画像密度を均等にする為、輪郭Ｇｒａｄｅのフィルタリングを使用する。またＸ線照射を制限する為、手動で照準する。

評価は下記の区分に従い判断する。

タイプ０　：　ストラットの破損無し

タイプⅠ ：　１つのストラットの破断のみ

タイプⅡ ：　１つのストラットの破断が複数箇所で発生

N タイプⅢ ：　複数のストラットの破断が発生しステントが切断されているが、ステントのマイグレーションはみられない。

タイプⅣ ：　複数のストラットの破断が発生し、ステントが部分的にマイグレーションしている＊

　　　　　＊タイプⅣには、ステントが切断されずマイグレーションすることがある「らせん状の破断」も含む。

付録５．　ABPIの測定方法

　　　　被験者を水平仰臥位で安静を保たせ、足関節部、内外果直上に腕用のカフを巻き、ドップラー血流計のプローブを足背動脈および後脛骨動脈上に置き、収縮期血圧を測定する。高い方の圧をもって、足関節部収縮期血圧（Ankle systolic pressure、ASP）とする。また、上腕に同様のカフを巻き肘窩にドップラー・プローブを置き、上腕収縮期血圧（Brachial systolic pressure、BSP）を測定する。

BSPに左右差がある場合には、高いほうをとって、ASP/BSP=ABPIを計算する。

付録６．　予想される有害事象（添付文書記載事項）

プレタール

**重大な副作用**

1. うっ血性心不全、心筋梗塞、狭心症（各0.1～5％未満）、心室頻拍（頻度不明※）

2出血＜脳出血等の頭蓋内出血（0.1～5％未満）＞＜肺出血（0.1％未満）、消化管出血、鼻出血、眼底出血（各0.1～5％未満）等＞

3. 胃・十二指腸潰瘍（0.1～5％未満）

4. 汎血球減少、無顆粒球症（いずれも頻度不明※）、血小板減少（0.1～5％未満）

5. 間質性肺炎（0.1％未満）

6. 肝機能障害（0.1～5％未満）、黄疸（頻度不明）

7. 急性腎不全（0.1％未満）

**その他の副作用**<0.1%以上または頻度不明の副作用記載>

1. 過敏症:発疹、皮疹、そう痒感,光線過敏症、紅斑

2.循環器: 動悸、頻脈、ほてり、血圧上昇、血圧低下、心房細動・上室性頻拍・上室性期外収縮・心室性期外収縮等の不整脈等

3. 精神神経系:頭痛・頭重感、めまい、不眠、しびれ感

4. 消化器 : 腹痛、悪心・嘔吐、食欲不振、下痢、胸やけ、腹部膨満感、味覚異常

5. 血液 : 貧血、白血球減少

6. 出血傾向 : 皮下出血、血尿等

7. 肝臓 : AST（GOT）・ALT（GPT）・Al-P・LDHの上昇等

8. 腎臓 : BUN上昇、クレアチニン上昇、尿酸値上昇、頻尿

9. その他 : 発汗、浮腫、胸痛、血糖上昇、耳鳴、倦怠感、結膜炎、発熱、脱毛

バイアスピリン

**重大な副作用**

1. ショック，アナフィラキシー様症状 （頻度不明）

2. 出血 （頻度不明）＜脳出血等の頭蓋内出血・肺出血，消化管出血，鼻出血，眼底出血等＞

3. 皮膚粘膜眼症候群（Stevens-Johnson症候群），中毒性表皮壊死症（Lyell症候群），はく脱性皮膚炎 （頻度不明）

4. ＊再生不良性貧血，血小板減少，白血球減少 (頻度不明）

5. 喘息発作 （頻度不明）

6. 肝機能障害，黄疸 （頻度不明）

7. 消化性潰瘍，小腸・大腸潰瘍 （頻度不明）

**その他の副作用**<0.1%以上または頻度不明の副作用記載>

消化器（胃腸障害，嘔吐，腹痛，胸やけ，便秘，下痢，食道炎，口唇腫脹，吐血，吐き気、悪心，食欲不振，胃部不快感）

過敏症（蕁麻疹, 発疹，浮腫）

皮膚（そう痒，皮疹，膨疹，発汗）

精神神経系（めまい，興奮,頭痛）

肝臓（AST（GOT）上昇，ALT（GPT）上昇）

循環器（血圧低下，血管炎, 心窩部痛）

呼吸器(気管支炎，鼻出血,鼻炎)

感覚器(耳鳴，難聴)

その他(過呼吸，代謝性アシドーシス,けん怠感)

プラビックス

重篤な副作用

1. 出血（頭蓋内出血、胃腸出血等の出血） ［脳出血等の頭蓋内出血（１％未満）、硬膜下血腫（0.1％未満）等］［吐血（頻度不明)）、下血、胃腸出血、眼底出血（いずれも１％未満）、関節血腫（0.1％未満）等］

2. 胃・十二指腸潰瘍（頻度不明)）

3. 肝機能障害、黄疸

4. 血栓性血小板減少性紫斑病（TTP)（頻度不明)）

5. 間質性肺炎（0.1％未満）

6. 血小板減少、無顆粒球症、再生不良性貧血を含む汎血球減少症（頻度不明)）

7. 中毒性表皮壊死融解症（Toxic Epidermal Necrolysis:TEN）、皮膚粘膜眼症候群（Stevens‐Johnson症候群）、多形滲出性紅斑（頻度不明)）

8. 横紋筋融解症（頻度不明)）

**その他の副作用**<0.1%以上または頻度不明の副作用記載>

血液 (皮下出血、貧血、紫斑（病）、鼻出血、止血延長、眼出血、歯肉出血、痔出血、血痰、穿刺部位出血、処置後出血、ヘモグロビン減少、赤血球減少、ヘマトクリット減少、白血球減少、好中球減少、好酸球増多,血清病)

肝臓 (Al‐P上昇、LDH上昇、血清ビリルビン上昇)

消化器 (消化器不快感、胃腸炎、口内炎、腹痛、嘔気、下痢、食欲不振、便秘、食道炎、嘔吐,大腸炎（潰瘍性大腸炎、リンパ球性大腸炎）、膵炎)

代謝異常 (中性脂肪上昇、CK(CPK)上昇、総コレステロール上昇、総蛋白低下、K上昇、アルブミン低下)

過敏症 (発疹、そう痒感、湿疹、蕁麻疹、紅斑,アナフィラキシー様反応、斑状丘疹性皮疹、血管浮腫、気管支痙攣)

皮膚 (水疱性皮疹、扁平苔癬)

精神神経系 (頭痛、高血圧、めまい,しびれ、筋骨格硬直（肩こり、手指硬直）、意識障害、不眠症、意識喪失、音声変調、低血圧、てんかん、眠気、皮膚感覚過敏、流涙、気分変動)

循環器 (浮腫、頻脈、不整脈,血管炎)

腎臓 (BUN上昇、血中クレアチニン上昇、尿蛋白増加、血尿、尿沈渣異常、尿糖陽性、腎機能障害,糸球体症)

その他 (ほてり、関節炎、発熱、異常感（浮遊感、気分不良）,筋痛、関節痛)

エフィエント

重篤な副作用

1. 出血（頭蓋内出血、胃腸出血等の出血） ［脳出血等の頭蓋内出血（１％未満）、硬膜下血腫（0.1％未満）等］［吐血（頻度不明)）、下血、胃腸出血、眼底出血（いずれも１％未満）、関節血腫（0.1％未満）等］

2. 胃・十二指腸潰瘍（頻度不明)）

3. 肝機能障害、黄疸

4. 血栓性血小板減少性紫斑病（TTP)（頻度不明)）

5. 間質性肺炎（0.1％未満）

6. 血小板減少、無顆粒球症、再生不良性貧血を含む汎血球減少症（頻度不明)）

7. 中毒性表皮壊死融解症（Toxic Epidermal Necrolysis:TEN）、皮膚粘膜眼症候群（Stevens‐Johnson症候群）、多形滲出性紅斑（頻度不明)）

8. 横紋筋融解症（頻度不明)）

**その他の副作用**<0.1%以上または頻度不明の副作用記載>

血液 (皮下出血、貧血、紫斑（病）、鼻出血、止血延長、眼出血、歯肉出血、痔出血、血痰、穿刺部位出血、処置後出血、ヘモグロビン減少、赤血球減少、ヘマトクリット減少、白血球減少、好中球減少、好酸球増多,血清病)

肝臓 (Al‐P上昇、LDH上昇、血清ビリルビン上昇)

消化器 (消化器不快感、胃腸炎、口内炎、腹痛、嘔気、下痢、食欲不振、便秘、食道炎、嘔吐,大腸炎（潰瘍性大腸炎、リンパ球性大腸炎）、膵炎)

代謝異常 (中性脂肪上昇、CK(CPK)上昇、総コレステロール上昇、総蛋白低下、K上昇、アルブミン低下)

過敏症 (発疹、そう痒感、湿疹、蕁麻疹、紅斑,アナフィラキシー様反応、斑状丘疹性皮疹、血管浮腫、気管支痙攣)

皮膚 (水疱性皮疹、扁平苔癬)

精神神経系 (頭痛、高血圧、めまい,しびれ、筋骨格硬直（肩こり、手指硬直）、意識障害、不眠症、意識喪失、音声変調、低血圧、てんかん、眠気、皮膚感覚過敏、流涙、気分変動)

循環器 (浮腫、頻脈、不整脈,血管炎)

腎臓 (BUN上昇、血中クレアチニン上昇、尿蛋白増加、血尿、尿沈渣異常、尿糖陽性、腎機能障害,糸球体症)

その他 (ほてり、関節炎、発熱、異常感（浮遊感、気分不良）,筋痛、関節痛)

付録7．匿名化番号対照表

匿名化番号対照表の使い方

臨床研究では、プライバシー保護の観点より、症例報告書には個人を特定できる情報は一切記載することが出来ません。全て登録番号で管理されます。

そのため、施設側で、患者個人と登録番号を対応させる為の管理が必要となります。

被験者識別コードは、個人情報を公表することなく患者を特定する為の唯一の情報となります。

※１　：　被験者識別コードは、個人情報を含まず、一意となる任意のコードをつけて下さい。

＜良くない例＞

ｙａｍａｔａｒｏ・・・・・・・・・・・・・・・・・・・・・山○太郎と推測可能です

　ＹＴ１９３７０３０４・・・・・・・・・・・・・・・・昭和12年3月4日生まれのイニシャルＹＴさんと推測可能です

ヤ２００５１２３４５・・・・・・・・・・・・・・・カルテ番号は個人を容易に特定できる為使用できません。

０７８３０３９１１７・・・・・・・・・・・・・・・・・患者の電話番号など、個人情報は使用できません。

症例報告書を作成する際には、かならずこの匿名化番号対照表で、患者のカルテと登録番号の対応をご確認の上、作成して下さい。

この表は、先生毎に作成して頂いても施設毎で管理して頂いても構いません。
