# Supplementary material for: Cilostazol effectiveness in reducing drug-coated stent restenosis in the superficial femoral artery: The ZERO study
Source: PLoS One. 2022 Jul 7;17(7):e0270992. doi: 10.1371/journal.pone.0270992 (PMC9262206; doi:10.1371/journal.pone.0270992)
Supplement: S1 Protocol — (DOC) [file pone.0270992.s003.doc]

ZilvER ptx with cilOstazol in Superficial femoral artery

ZERO

Study protocol

Principal Investigator

Nagano Municipal Hospital Department of Cardiology

Takasi Miura

Research Advisor:

Nagano Red Cross Hospital, Department of Cardiology

Yusuke Miyashita

Created date

April 23, 2018 Draft plan 2nd edition created

Confidentiality statement:

This research implementation plan must not disclose information to anyone other than those directly involved in this research and the Institutional Review Board. In addition, this information shall not be used for any purpose other than the implementation or evaluation of this research without the prior written consent of the principal investigator.

Rules to be observed:

Everyone involved in this study follows the World Medical Association Declaration of Helsinki and the Ethical Guidelines for Clinical Research.

○ Purpose

To investigate the effect of cilostazol on restenosis prevention for drug-eluting stents in femoral popliteal artery region lesions

○ Main eligibility criteria

 Patients with chronic arteriosclerosis obliterans with Rutherford classification 2-4 lesions in the femoral popliteal artery region. However, acute (within 7 days) and subacute (within 1 month) lower limb ischemia cases are excluded.

 Age: Must be at least 20 years old at the time of obtaining consent. Gender: No matter

 Patients who can be observed for 12 months or more (Patients who can follow up after 12 months)

○ Target number of cases

90 cases: Zilver-PTX + cilostazol group 90 cases

○ Registration period

April 2018-End of March 2019

○ Research period

April 2018-end of March 2020

○ Research design

Multicenter prospective study

○ Research evaluation items

Main endpoint

1. Primary Patency (PSVR ≤ 2.0): Avoidance from restenosis of target lesions

Secondary endpoint

2. Bleeding complications (cerebral hemorrhage, gastrointestinal hemorrhage, etc.)

3. TVR (Target Vessel Revascularization): Revascularization of target blood vessels

4. MALE (Major Adverse Limb Event): Lower limb-related death + amputation of lower limb + TLR + bleeding complications

5. Stent breakage rate

6. Cardiovascular event

7. Adverse events

○ Contact information

Research inquiries

Research Secretariat

Shinshu University School of Medicine, Endovascular Surgery Course

Person in charge: Kato Taimon Kurihara Maki

3-1-1 Asahi, Matsumoto City, Nagano Prefecture 390-0802

TEL: 0263 (37) 3486 (representative) FAX: 0263 (37) 3489

table of contents

1. 1. Purpose

2. Background and rationale

3. 3. Criteria / definitions used in this study

3-1. Cardiovascular event

3-2. Lower limb vascular event

3-3. Adverse events

3-4. All deaths

3-5. Restenosis after stent placement

3-6. Retreatment after stent placement

Four. Eligibility criteria

4-1. Selection criteria

4-2. Exclusion criteria

Five. Survey method and research schedule

5-1. Patient consent

5-2. Registration method

5-3. Research design

5-4. Research agents and usage

5-5. Revascularization

5-6. Survey / inspection item schedule

6. Survey / inspection items and implementation methods

6-1. Regulations on implementation time

6-2. Patient background

6-3. Survey items regarding endovascular treatment of lower limbs

6-4. Administration status of research drugs

6-5. Ischemia symptoms

6-6. Ankle Brachial Pressure Index (ABPI)

6-7. Stent breakage

6-8. Clinical examination

6-9. Cardiovascular events

6-10. Lower limb vascular event

6-11. Adverse events

6-12. Expected adverse events

7. 7. cancel

8. Target number of cases and research institute

8-1. Target number of cases

8-2. Research period

9. Endpoint definition and safety endpoints

9-1. Main endpoint

9-2. Secondary endpoint

9-3. Safety evaluation items

10. Statistical consideration

10-1. Basis for setting the target number of cases

10-2. Population to be analyzed

11. Case report

11-1. Style

11-2. Input method (or description)

11-3. Input (or description) of case report form Confirmation and inquiry

12. Ethical matters

12-1. Rules to be observed

12-2. Preparation and revision of explanatory documents and consent forms (forms)

12-3. Protection of personal information

13. Protocol approval and revision

13-1. Protocol approval

13-2. Revision of protocol

14. End of study and early discontinuation

14-1. End of research

14-2. Early discontinuation of research

15. Keeping records

16. Burden of research costs

16-1. Conflicts of funding sources and possible interests

16-2. Compensation for treatment costs and health hazards

17. Research organization

18. Announcement of results

19. Literature

20. Imprint

List of abbreviations

EVT　　　Endovascular　　Therapy

PTA　　　Percutaneous　　transluminal　angioplasty

TIA　　　Transient　ischemic　attack

ACD　　　Absolute　　claudication　　distance

PSV　　　Peak Systolic Velocity

1. Purpose

Comparing the effect of cilostazol on restenosis prevention for drug-eluting stents in femoral popliteal artery region lesionsこと

。

２．Background and rationale

In recent years, the number of arteriosclerosis obliterans of the lower extremities has increased remarkably with the increase of arteriosclerosis diseases. Arteriosclerosis obliterans of the lower extremities is a partial disease of systemic arteriosclerosis vascular lesions, and ischemic complications of important organs are often observed. Lower limb endovascular treatment is about to be selected. In the iliac artery region, endovascular treatment of the lower extremities using a stent has been established. However, in superficial femoral artery lesions, although the results of lower limb vascular treatment have been dramatically improved by introducing a stent, the long-term patency rate is not sufficient. It has long been pointed out that the cause is neointimal hyperplasia, which is mainly composed of fibroblasts and smooth muscle cells, and even in the Trans Atlantic Inter-Society Consensus (TASC) announced in 2000, after revascularization. As an adjunct therapy to, the need to investigate the clinical efficacy of substances reported to inhibit intimal hyperplasia is of concern.

Nanto et al. Reported the effect of cilostazol on preventing restenosis in a retrospective analysis of 121 lesions in the femoral popliteal artery region, and a follow-up study of 127 patients in a prospective study for up to 3 years. It was reported that the patency rate was significantly higher in the cilostazol group than in the ticlopidine group. Furthermore, it is said that the progression of restenosis is highest one year after EVT, but it became clear that the cilostazol group suppressed restenosis especially in the first year. (1) In addition, Iida et al. Found the restenosis rate of the cilostazol group (cilostazol + aspirin) and the non-cilostazol group (aspirin alone) in 151 patients with first-generation nitinol stents placed in the femoral patellar artery region. The analysis reported that the 12-month restenosis rate was 21% in the cilostazol group and 48% in the non-cilostazol group. (2) Furthermore, Miyashita et al. In TCT (Transcatheter Cardiovascular Therapeutics) 2017, a group of 270 patients who used a second-generation nitinol stent (Misago stent) under normal antiplatelet drug use, and a group with cilostazol in combination. One-year restenosis analysis was performed for 3 groups, a group using a 2nd generation nitinol stent (Misago stent) and a group using a drug-eluting stent (Zilver PTX) using aspirin and Plavix, and analyzed 29.1% and 11.4. %, 21.3% was reported (DEBATE in SFA study). In addition, all of them performed a retrospective analysis of the Zilver PTX prospective registry (ZEPHYR) to analyze the one-year restenosis by adjusting the background using propensity matching in the group that received Zilver PTX siloszol and the group that did not. It was reported that it was 31% and 51%. (3)

That is, many studies have demonstrated the restenosis-preventing effect of cilostazol on first- and second-generation nitinol self-stents. However, the preventive effect of cilostazol on drug-eluting stents for restenosis has not been positively proven.

Based on the above, a single group using a drug-eluting stent in combination with cilostazol was prospectively registered, and the DEBATE in SFA data was used as historical data to compare the efficacy and safety of the superficial femoral artery region. Consider further optimal endovascular treatment.

3. Criteria / definitions used in this study

3-1. Cardiovascular event

It shall correspond to any of ischemic stroke including TIA, hemorrhagic stroke, myocardial infarction, and other vascular accidents (see Appendix 2 for diagnostic criteria here).

3-2. Lower limb vascular event

Any of amplification (major / minor), transition to bypass surgery, revascularization (including TLR *, TVR **, revascularization other than responsible vessels), stent thrombosis, lower limb-related death, and bleeding complications It shall correspond to.

* TLR (Target Lesion Revascularization): Revascularization of target lesions

** TVR (Target Vessel Revascularization): Revascularization of the target blood vessel.

Both TLR and TVR will be implemented clinically driven.

If you plan to carry out EVT for both limbs at the time of obtaining consent, the later EVT will not be treated as an event.

3-3. Adverse event

All events that occur during the study period that are detrimental to the patient (including abnormal changes in laboratory test values), regardless of the relationship with the research drug. In this study, "3-1. Cardiovascular event" is a secondary endpoint, and "3-2. Lower limb vascular event" is a major endpoint, but it is not an adverse event.

3-4. All deaths

All deaths regardless of the reason for death. (Refer to Appendix 3 for the breakdown of causes of death.)

3-5-1. Restenosis after stent placement

Restenosis: Angiography confirms stenosis of 50% or more (Binary restenosis), or lower limb echo with PSVR> 2.0.

3-5-2. Definition of stented blood vessels

The area from the healthy part to the healthy part on both sides of the site where the stent was placed

3-6. Retreatment after stent placement

Endovascular treatment for the purpose of treatment performed on the same blood vessel after endovascular treatment, bypass surgery is defined as retreatment.

1. 1. Eligibility criteria

4-1. Selection criteria

4-1-1. Patient selection criteria

Patients who meet all of the following conditions will be studied.

1. 1. Patients with chronic arteriosclerosis obliterans with femoral popliteal artery region lesions (Rutheford classification 2-4).

However, cases of acute (within 7 days) and subacute (within 1 month) lower limb ischemia are excluded.

2. Age: 20 years or older (at the time of consent)

3. 3. Gender: No matter

Four. Patients who can be observed for 12 months or more after surgery (Patients who can follow up 12 months later)

4-1-2. Lesion selection criteria

1. 1. Angiography shows significant stenosis or occlusion of the superficial femoral artery and is limited to new lesions. The lower pole of the superficial femoral artery is the part that overlaps the bone of the adductor longus muscle in the upper part of the femur, and the upper pole is the origin of the bifurcation.

2. It is possible to have one or more run-offs of the inferior artery and stenotic lesions without flow limiting. In addition, patients with bilateral lesions and patients with aortic-iliac artery lesions are also included. However, in patients with bilateral lesions, endovascular treatment should be performed at intervals of 30 to 45 days for each limb.

3. 3. Obstructive lesions are also included.

4-2. Exclusion criteria

4-2-1. Patient exclusion criteria

Patients who meet any of the following conditions are excluded from the study.

1. 1. Patients who are bleeding or have a tendency to bleed

(Hemophilia, capillary fragility, intracranial hemorrhage, gastrointestinal hemorrhage, urinary tract bleeding, hemoptysis, vitreous hemorrhage, etc.)

2. Patients with severe congestive heart failure

3. 3. Patients who cannot reduce the dose of antiplatelet drug alone after implanting DES (Drug Eluting Stent) into the coronary artery

4. Patients taking anticoagulants (warfarin, pradaxa, ixarelto)

5. Patients with a history of serious side effects or hypersensitivity to the components of the research drug

6. Patients who are pregnant or may be pregnant

7. Patients with acute / subacute lower limb ischemia

8. Other patients who are considered inappropriate for research at the discretion of the attending physician

4-2-2. Lesion exclusion criteria

Lesions that meet any of the following conditions are excluded from the study.

1. 1. Inflow (aorta-iliac artery lesion) lesion remains. However, if treatment is performed at the same time, it may be the subject of research.

2. Less than one run-off of the inferior artery

5. Survey method and research schedule

5-1. Patient consent

The research-responsible (in charge) doctor shall explain the following contents to the patient himself / herself and obtain written consent from the patient himself / herself regarding participation.

① Purpose of research

② Research method

③ Expected clinical benefits and risks

④ Presence or absence of other treatment methods

⑤ Even if you do not agree to participate in the research, you will not be disadvantaged.

⑥ Participation in research can be withdrawn at any time

⑦ Conditions or reasons for cancellation

⑧ The cost burden when participating in the research is within the scope of normal insurance medical treatment.

⑨ Presence or absence of compensation for research

⑩ Privacy must be protected.

⑪ This study has been approved by the Institutional Review Board.

⑫ Research period and number of participants

⑬ Research schedule

⑭ Contact information for inquiries, etc.

5-2. How to register

The principal investigator applies to the ethics review committee of each institution for approval before conducting the research.

After obtaining approval from the ethics review board of each institution, the investigator (in charge) will send a certificate of passing the ethics committee to the registration center by email.

Case registration and administration of research agents shall be in accordance with the Registration Center Manual.

Registration Center Registration Secretariat

Shinshu University School of Medicine, Endovascular Surgery Course

3-1-1 Asahi, Matsumoto City, Nagano Prefecture 390-0802

TEL: 0263 (37) 3486 (representative) FAX: 0263 (37) 3489

Person in charge Maki Kurihara (junkan@shinshu-u.ac.jp)

５－３．Study design


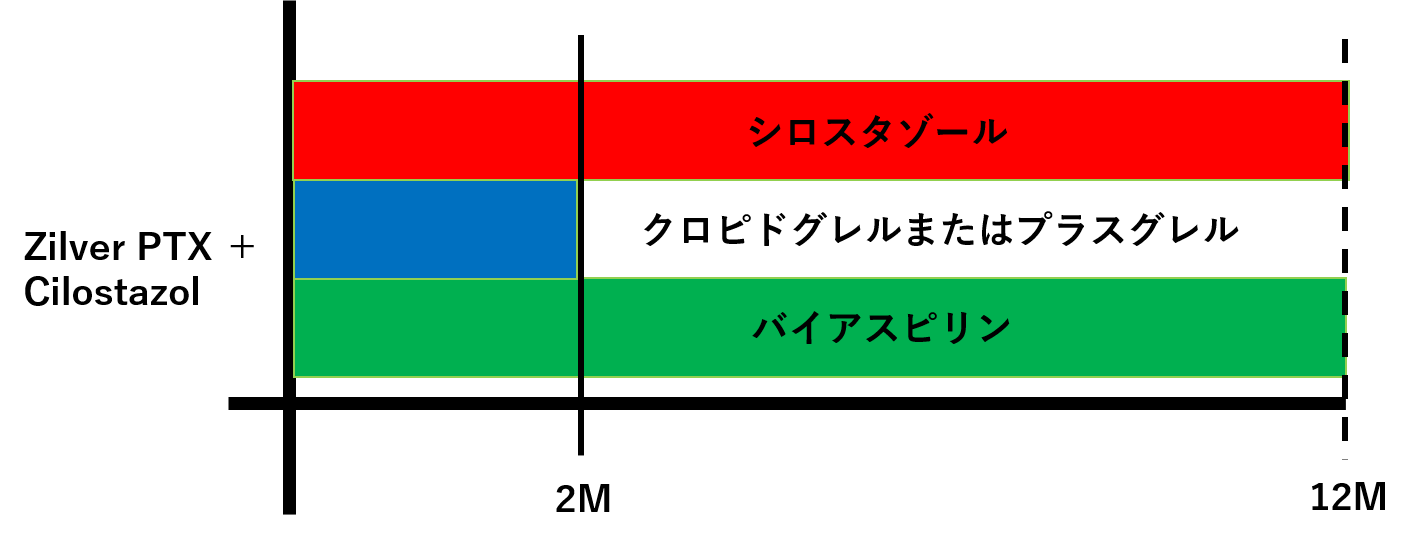


Cilostazol

Clopidogrel or prasugrel

Aspirin

5-4. Research drug and usage

Aspirin should be 100 mg / 1, clopidogrel should be 75 mg / 1, prasugrel should be 3.75 mg / 1, and cilostazol should be 200 mg / 2. Oral administration should be started 3 to 7 days or more before the implementation of EVT. Discontinue clopidogrel or effient 2 months after EVT.

If the dose must be reduced due to side effects such as headache after the start of cilostazol administration, the dose can be reduced to 100 mg / 2 per day.

2. Concomitant drug

As a general rule, antiplatelet drugs other than the following aspirin, clopidogrel, prasugrel, and cilostazol should not be used during the study period, but if unavoidable, they can be used in combination, but the reason is described in the doctor's comment section. do. Ticlopidine hydrochloride (panaldine) will be changed to clopidogrel.

5-5. Revascularization

Any approach can be used for revascularization.

The size should be 4 mm or more with the reference diameter of the target blood vessel. The length of the lesion should be less than 36 cm. Stent indication is 10 mmHg or% DS> 30% pressure gradient after balloon dilation, Flow Limiting dissection.

In this clinical study, if EVT is planned for both limbs at the time of consent acquisition, the period of EVT for both limbs shall not exceed 30 to 45 days. For the observed limbs in this study, the limbs that underwent EVT first will be registered, and the EVT of the limbs that were performed later will not be treated as an event.

5-6. Survey / inspection item schedule

[Observation items and observation time]

|  |  | 定期検査（同意取得後） | | | | | |
| --- | --- | --- | --- | --- | --- | --- | --- |
| factor／piriod | before | after | １M | ３M | ６M | １２M | When administration is discontinued or an event occurs |
| Patient background | ● |  |  |  |  |  |  |
| Ischemia symptoms | ● |  | ○ | ● | ● | ● | ● |
| Laboratory test values | ○ |  | ○ | ○ | ● | ● | ● |
| Events　※２ |  | | | | | | |
| Adverse event |  | | | | | | |
| Device information　※１ |  | ● |  |  |  |  | ● |
|  |  |  |  |  |  |  |  |

●; Required, ○ Non-essential (However, clinical tests should be conducted as much as possible)

* 1: Evaluation of stent breakage rate by X-ray

* 2: Events include cardiovascular events, lower limb vascular events, and all deaths.

† Entry is permitted for cases that have passed more than one month after DES placement and that the attending physician has determined that the dose can be reduced to an antiplatelet drug alone.

6. Survey / inspection items and implementation methods

6-1. Regulation of implementation time

The definition of the timing of each survey is as follows.

| factor | definition |
| --- | --- |
| after | After endovascular treatment of the lower limbs, until leaving the treatment room |
| 1 month after surgery | 1 month ± 2 weeks immediately after surgery |
| 3 month after surgery | 3 months ± 1 month immediately after surgery |
| 6 month after surgery | 6 months ± 1 month immediately after surgery |
| 12 month after surgery | 12 months ± 1 month immediately after surgery |

6-2. Patient background

Basic information

□ Subject identification code (see "Appendix 7. Anonymization Number Comparison Table")

□ Treatment date

□ Age at treatment

□ Gender

□ Concomitant drug name, dosage, start date, end date

□ Height, weight, age

□ Rutherford classification

Medical history / complications / smoking rate

□ Hypertension: Yes, No

□ Bleeding: Yes, No

□ Diabetes: Yes, No

□ Dyslipidemia: Yes, No

□ Coronary artery disease: Yes, no

□ Stroke / TIA history: Yes, No

□ Smoking history: Yes, No

□ Dialysis: Yes, No

Definition of medical history / complications

| Medical history / complications | Definition |
| --- | --- |
| Hypertension | Those who meet either systolic blood pressure of 140 mmHg or higher, diastolic blood pressure of 90 mmHg or higher, or oral antihypertensive drug for the purpose of lowering blood pressure at any medical examination within 3 months before endovascular treatment. |
| Bleedind | History of bleeding requiring hospitalization or blood transfusion |
| Diabetes mellitus | Fasting blood glucose 126 mg / dL or higher or occasional blood glucose 200 mg / dL or higher or oral hypoglycemic drug, administration of insulin preparations, and previously diagnosed diabetes at a medical institution at any medical examination within 3 months before intravascular treatment. , Those that meet any of these. |
| Dyslipidemia | Those who meet LDL cholesterol ≥140 mg / dL, HDL cholesterol <40 mg / dL, torglyceride ≥150 mg / dL, or oral lipid-improving drug at any of the medical examinations within 3 months before endovascular treatment. |
| Coronary artery disease | Those who have undergone acute myocardial infarction, angina, coronary angioplasty, or coronary artery bypass grafting in the past. |
| History of stroke / TIA | Those who have been treated for cerebral infarction, cerebral hemorrhage, subarachnoid hemorrhage or TIA in the past. |
| Smoking history | Those who have an average smoking habit of 1 cigarette / day or more within 1 year before endovascular treatment. |

6-3. Survey items for endovascular treatment of lower limbs

Lower limb endovascular treatment information (including device information)

□ Treatment foot (for each procedure)

: Left, right

: Type (TASC classification * A, B, C, D)

* TASC classification is based on TASC II

: Vascular diameter (mm), length (mm), stenosis rate (%) Please attach a ruler at the time of treatment.

: Run off state (number 1, 2, 3)

□ Lesions of the inferior arteries (tibialis anterior, posterior cervical, and peroneal arteries) * Site

* Stenosis or closure with decreased blood flow

□ Device type

Stents, balloons, etc.

□ Stent diameter (mm)

□ Stent length (mm)

□ Number of stents ()

□ Front expansion: Maximum balloon diameter (mm)

□ Post-expansion: Maximum balloon diameter (mm)

□ Onset of complications associated with the procedure: Yes, No

<If there is>

□ Contents: Bleeding, hematoma, blood transfusion, peripheral embolism, fat, lower limb amputation, emergency surgery

□ Peripheral embolism: Yes, No, Date of onset

□ Hemorrhagic complications: Yes, no (blood transfusion, bleeding at the puncture site requiring surgical repair), date of onset

6-4. Administration status of research drugs

□ Cilostazol (start date, end date, daily dose)

□ Aspirin (start date, end date, daily dose)

□ Clopidogrel sulfate (start date, end date, daily dose)

6-5. Ischemia symptoms

□ Cooling sensation:-, ±, +, ++, +++

□ Numbness:-, ±, +, ++, +++

□ Intermittent claudication: Rutherford classification

Evaluate with.

6-6. Ankle Brachial Pressure Index (ABPI) ratio

□ ABPI

(Recommended by the Intermittent Claudication Severity Assessment Subcommittee of the Japanese Society of Angiology)

- 6-7. Stent breakage
- □ Check the presence or absence of strut breakage by X-ray photography of the stent, and judge the degree of breakage with types 0 to IV.

6-8. Vascular echo

Lesion PSV, Lesion PSVR

6-9. Laboratory test

① Hematological examination

Red blood cell count, white blood cell count, platelets, Hb, Ht

② Biochemical test

Total protein, albumin, total bilirubin, AST (GOT), ALR (GPT), Al-p, γ-GTP, BUN, creatinine, LDL cholesterol, triglyceride, HDL-cholesterol, fasting blood glucose, HbA1c, CRP

　　6-10. Cardiovascular events and all-cause mortality

□ Yes, No

□ Event onset date / final follow-up confirmation date

If "Yes", enter the date of onset of the first event, and if "No", enter the final follow-up confirmation date.

≪In the case of "Yes" ≫

□ Event content

Ischemic stroke including TIA, hemorrhagic stroke, myocardial infarction, other vascular accidents (aortic dissection or rupture, pulmonary embolism, or limb infarction) and all-cause mortality (stroke death, cardiovascular, etc. unknown). If multiple events occur, describe the date when the first event occurred and the confirmed event.

6-11. Lower limb vascular event

□ Yes, No

□ Event onset date / final follow-up confirmation date

If "Yes", enter the date of onset of the first event, and if "No", enter the final follow-up confirmation date.

<In the case of "Yes">

□ Event details: Amption (major / minor), transition to bypass surgery, revascularization (including TLR *, TVR **, revascularization other than responsible blood vessels), stent thrombosis, lower limb-related death, bleeding complications

If multiple events occur, describe the date when the first event occurred and all the confirmed events.

6-12. Adverse event

During the study period, all abnormal findings (including death) that were not diagnosed before the administration of the research drug and abnormal changes in clinical laboratory test values ​​were observed. However, cardiovascular events and lower limb vascular events are not included.

6-13. Expected adverse events

See Appendix 6 for expected adverse events related to research drugs

7. cancel

In the following cases, the continuation of research on that case will be discontinued.

1) Withdrawal of patient consent

2) When the patient stops coming to the hospital for unknown reasons

3) When the doctor responsible for research (in charge) determines that it is difficult to continue other than 2)

4) Completion of the entire research

2. Target number of cases and study period

8-1. Target number of cases

90 cases

8-2. Research period

From April 2018 to the end of March 2020 (The case registration period is from April 2018 to the end of March 2019.)

3. 3. Endpoint definition and safety endpoints

9-1. Main endpoint

Primary Patency: Avoidance from restenosis of target lesion (avoidance from PSVR ≥ 2.0 in vascular ultrasound)

MALE (Major Adverse Limb Event): Lower limb-related death + amputation of lower limb + TLR + bleeding complications

9-2. Secondary endpoint

① Bleeding complications (cerebral hemorrhage, gastrointestinal hemorrhage, etc.)

(2) TVR (Target Vessel Revascularization): Revascularization of target blood vessels

③ MALE (Major Adverse Limb Event): Lower limb-related death + amputation of lower limb + TLR + bleeding complications

④ Stent breakage rate

⑤ Cardiovascular event

⑥ Adverse event

9-3. Safety evaluation items

1. Discontinuation of the drug

② Adverse event

10. Statistical consideration

10-1. Basis for setting the target number of cases

Twice

Miyashita et al. In TCT (Transcatheter Cardiovascular Therapeutics) 2017, in the femoral popliteal artery region

A group of 270 patients who used a second-generation nitinol stent (Misago stent) under normal antiplatelet drug use,

Group using 2nd generation nitinol stent (Misago stent) in combination with cilostazol, aspirin, plavit

We analyzed restenosis in 3 groups using drug-eluting stents (Zilver PTX) under the use of drugs, and reported as follows.

doing. The restenosis rate at 12 months was 29.1% in the 2nd generation nitinol stent group under non-cilostazol, and cilosta.

2nd Generation Nitinol Stent Group with Oral Zol, 11.4%, Drug-eluting Stent Group with Non-Cilostazol

It is 21.3%. In this study, 90 cases per group were registered and analyzed. Use this data as historical data

Therefore, the target case is set to 90 cases in this study as well.

Twice

10-2. Population to be analyzed

In this study, all registered patients who meet the eligibility criteria will be included in the analysis target population.

Twice

11. Case report form

11-1. Style

The case report (CRF) is a document issued by the research office and describes the data. It also includes printouts after data entry.

11-2. Input method (or description)

Observe the following items when entering the case report form.

・ ・ Input and correction will be done by the research-responsible (in charge) doctor.

・ When inputting, refer to "Appendix 7. Anonymization Number Comparison Table" and confirm that the medical record belongs to the subject.

・ After completing the data entry, print out the case report form and keep it in a medical record.

・ If you have any questions about the input method, contact the research office.

11-3. Input (or description) of case report form Confirmation and inquiry

After entering the data, the research secretariat will print it out and store it in a medical record, etc.

・ Insufficient input

・ Protocol integrity

・ Consistency between input contents of case report form

The research secretariat summarizes the points to be inquired in a data inquiry form and sends it to the investigator.

The research responsibility (doctor) will make input and correction or fill in the answer on the data inquiry form, reply to the research secretariat, and keep it until the principal investigator notifies the end of the research.

12. Ethical matters

12-1. Rules to be observed

All persons involved in this study are the "World Medical Association Declaration of Helsinki (revised in October 2002)" and "Ethical Guidelines for Clinical Research (Ministry of Education, Science and Technology, Ministry of Health, Labor and Welfare, December 22, 2014 (February 2017)" Conduct research in compliance with (partially revised on 28th of March).

12-2. Creation and revision of explanatory documents and consent forms (forms)

The explanation document, consent form (form) and consent withdrawal form are created by the research responsible doctor. However, the "Explanatory document / consent form (form) created by the principal investigator may be modified and used. The prepared explanatory document / consent form (form) is the ethics of the medical institution to which the research belongs before the start of the research. Submit it to the review committee for approval.

‥

If the research-responsible (in charge) doctor obtains new knowledge related to the consent of the subject after the start of the research and determines that the explanatory document / consent form (form) needs to be revised, it will be revised. The new findings related to the consent of the subject refer to, for example, information on new adverse events related to the treatment method, information on the development of new treatment methods related to the disease, and the like. If the content of the revision is judged to be serious, submit it to the ethics review committee of the medical institution to which you belong and obtain its approval.

12-3. Protection of personal information

The original medical record can be viewed by the Institutional Review Board and those involved in the research.

Persons involved in the research will make the utmost efforts to protect the personal information of the subjects.

When providing a case registration slip, case report, etc. outside the medical institution, the research-responsible (in charge) doctor attaches a new subject identification code and uses it in order to perform linkable anonymization. Information that allows a person outside the medical institution to identify the subject (name, address, telephone number, etc.) is not included.

Subject identification managed by the research responsible (charge) doctor is the identification of the subject when the registration center inquires to the medical institution.

Do it with code.

When the principal investigator, etc. publishes the information obtained in the research, give due consideration so that the subject cannot be identified.

13. Protocol approval and revision

13-1. Protocol approval

In this research, the protocol will be examined by the Institutional Review Board of the research participating facility or an organization equivalent to it, and after approval, the facility registration of the research participating facility will be carried out. After the facility registration is completed, the research will be conducted at the participating facilities.

13-2. Protocol revision

When it becomes necessary to revise the protocol after the start of this research, it will be decided to discontinue or continue the research according to the content of the revision, and the research secretariat will notify each research participating facility to that effect.

The change of the protocol will be decided by the protocol review committee (members: principal investigator, deputy principal investigator, statistical analysis manager, and other necessary members) invited by the principal investigator. The examination results shall be submitted to the Nagano Municipal Hospital Ethics Review Committee for approval. In addition, after obtaining the approval of the Institutional Review Board of the research participating facility or an organization equivalent to it, the research will be conducted and resumed at the research participating facility under a new protocol.

14. End of research and early discontinuation

14-1. End of research

The research will be completed with the database fixed, and the principal investigator will report to that effect to the responsible doctor and statistical analysis manager of this research participating facility.

14-2. Early discontinuation of research

If it becomes necessary to discuss the pros and cons of continuing research, the principal investigator will discuss with the efficacy and safety evaluation committee and the person in charge of statistical analysis.

15. 15. Saving records

The principal investigator, the principal investigator (in charge), and the research secretariat will keep records (documents and electronic records) related to the implementation of this research for three years after the completion of the research.

16. Burden of research costs

16-1. Conflicts of funding sources and possible interests

This research is carried out with the support of the Department of Cardiovascular Medicine, Shinshu University School of Medicine (Internal Medicine No. 5). Regardless of the results of this study, it will not cause any benefit or damage to the Department of Cardiovascular Medicine (Internal Medicine No. 5), Shinshu University School of Medicine.

16-2. Compensation for treatment costs and health hazards

This research is conducted within the scope of normal health insurance, and the subject's health insurance is applied to observations / tests, drugs used, etc. related to the research. In addition, the treatments performed in this study are generally performed in ordinary medical examinations, and there is no risk that exceeds those in ordinary medical examinations.

17. Research organization

Principal researcher

Takashi Miura, Department of Cardiology, Nagano Municipal Hospital

1333-1 Tomitake, Nagano City, Nagano Prefecture, 381-0006 〒381-0006

TEL: 0262 (95) 1199 (representative) FAX: 0262 (95) 1148

Deputy Chief Researcher

Koji Parkzawa, Department of Cardiology, New Tokyo Hospital

1271 Wanagaya, Matsudo City, Chiba Prefecture 270-2232

TEL: 047-711-8700

Kato Taimon, Department of Cardiology, Shinshu University Hospital

3-1-1 Asahi, Matsumoto City, Nagano Prefecture 390-8621

TEL: 0263 (37) 3486 (representative) FAX: 0263 (37) 3489

Research advisor

Yusuke Miyashita, Department of Cardiology, Shinshu University Hospital

3-1-1 Asahi, Matsumoto City, Nagano Prefecture 390-8621

TEL: 0263 (37) 3486 (representative) FAX: 0263 (37) 3489

Yoshimitsu Soga, Department of Cardiology, Kokura Memorial Hospital

3-2-1 Asano, Kokurakita-ku, Kitakyushu-shi, Fukuoka 802-8555

TEL: 093 (511) 2000 (representative)

Research Assistant

Masaki Kurihara, Department of Cardiology, Shinshu University Hospital

3-1-1 Asahi, Matsumoto City, Nagano Prefecture 390-8621

TEL: 0263 (37) 3486 (representative) FAX: 0263 (37) 3489

Efficacy and safety assessment committee

Juntendo University School of Medicine, Department of Cardiology, Kasai Takatoshi

18. Announcement of results

The Protocol Review Committee is responsible for submitting and publishing research results. It is also responsible for the confidentiality of research participants.

19．　文献

１） Iida O, Nanto S et al.: J Vasc Surg. 2008; 48(1):144-9

2) Iida O, et al.: Circulation. 2013; 127: 2307-2315

3) Zen K, et al.: J Vasc Surg. 2017; 65: 720–5.

Appendix 1. Rutherford classification

[Rutherford RB et al: Suggested standards for reports dealing with lower extremity ischemia. J Vasc Surg 26: 517, 1997]

|  |  | Clinical definition | Objective criteria |
| --- | --- | --- | --- |
| 0 | 0 | Asymptomatic, no significant obstructive lesions in terms of hemodynamics | Normal treadmill exercise test or reactive hyperemia test |
|  | 1 | Mild claudication | Treadmill exercise load test can be completed (*), AP after exercise> 50 mmHg, but at least 20 mmHg lower than at rest |
| I | 2 | Moderate claudication | Between groups 1 and 3 |
|  | 3 | Severe claudication | Unable to complete standard treadmill exercise stress test (*) and AP <50 mmHg after exercise |
| II | 4 | Ischemic resting pain | Resting AP <40mmHg, flattening of ankle or metatarsal PVR or drastic decrease in wave height TP <30mmHg |
| III | 5 | Mild tissue loss, non-healing ulcer, localized gangrene with late foot ischemia | Resting AP <60mmHg, flattening of ankle joint or metatarsal PVR or drastic decrease in wave height TP <40mmHg |
|  | 6 | Extensive tissue loss expanded to a higher level than TM, no longer functional foot rim salvage | Same as group 5 |

Appendix 2. Cardiovascular event diagnostic criteria

Ischemic stroke

Neurological dysfunction with the report of Wholey et al. [10], transient cerebral ischemic attack (TIA), minor neurological dysfunction (remission within 7 days or worsening of NIH stroke scale <4 points) Is classified as minor stroke, and those whose neurological dysfunction persists after 7 days and worsens to NIH stroke scale of 4 points or more are classified as major stroke.

Transient ischemic attack (TIA)

In this study, the diagnostic criteria for TIA are "those with a duration of neurological symptoms of less than 24 hours (excluding those thought to be due to intraoperative blood circulation blockage by the CAS procedure)" regardless of the presence or absence of imaging findings. TIA should not be diagnosed with symptoms such as impaired consciousness or convulsions without neurological symptoms, scintillating scotoma, or single dizziness, diplopia, or dysarthria. The presence or absence of image findings is not used as a reference.

Hemorrhagic stroke

Symptomatic subarachnoid hemorrhage and intracerebral hemorrhage diagnosed by CT or MRI. This is not the case if the symptoms are headache only and not accompanied by other objective neurological symptoms.

Myocardial infarction

Diagnose myocardial infarction by any of 1 to 4 shown below according to the AHA / ACC guidelines. However, those diagnosed with clinical myocarditis and pericarditis are excluded.

1. 1. Appearance of new abnormal Q wave

2. Has typical or atypical chest symptoms, has ischemic electrocardiographic changes, and has elevated myocardial deviation enzymes << Elevated CK (CPK) or CK-MB (CPK-MB) more than twice the institutional standard >> Accompanied by

3. 3. With typical chest symptoms and elevated myocardial escape enzymes

4. Type autopsy findings (histological fresh infarction or recent coronary occlusion findings)

Other vascular accidents

Aortic dissection or rupture, pulmonary embolism, organ or limb infarction.

Appendix 3. All deaths

Stroke death: Death from ischemic stroke, hemorrhagic stroke

Cardiovascular death: Death from myocardial infarction and other vascular accidents

Other deaths: All deaths due to other causes

Lower limb-related death: Death due to vascular accident or infection in the lower limbs

Unknown: The cause of death is not clear

Appendix 4. Stent damage

The resolution should be the highest possible resolution so that the struts of the entire stent can be seen by X-ray photography.

In addition, the judgment is made based on both the stents at the time of flexion and extension of the lower limbs, and the front image without missing the stent image and the overlap with the bone and the images taken from two directions at right angles to the front image. For the image, a high-quality screen (when using film) or an equivalent histogram (computer X-ray photography: CR) shall be used. In addition, contour grade filtering is used to equalize the image density. Also, aim manually to limit X-ray irradiation.

Evaluation is judged according to the following categories.

Type 0: No strut damage

Type I: Only one strut breaks

Type II: One strut breaks at multiple locations

N type III: Multiple struts ruptured and the stent was cut, but no stent migration was observed.

Type IV: Multiple struts are broken and the stent is partially migrated *

* Type IV also includes "spiral breakage" in which the stent may migrate without being cut.

Appendix 5. ABPI measurement method

Keep the subject in a horizontal supine position, wrap an arm cuff just above the ankle joint, medial and lateral malleolus, and place a Doppler blood flow meter probe on the dorsalis pedis and posterior tibial arteries to measure systolic blood pressure. .. The higher pressure is used as the ankle systolic pressure (ASP). In addition, a similar cuff is wrapped around the upper arm and a Doppler probe is placed in the cubital fossa to measure brachial systolic pressure (BSP).

If there is a difference between the left and right BSP, take the higher one and calculate ASP / BSP = ABPI.

Appendix 6. Expected adverse events (items to be stated in the package insert)

Pletal

Serious side effects

1. Congestive heart failure, myocardial infarction, angina (less than 0.1-5% each), ventricular tachycardia (incidence unknown *)

2 Bleeding <Cranial hemorrhage such as cerebral hemorrhage (less than 0.1-5%)> <Pulmonary hemorrhage (less than 0.1%), gastrointestinal hemorrhage, epistaxis, fundus hemorrhage (less than 0.1-5% each)>

3. Gastric / duodenal ulcer (less than 0.1-5%)

4. Pancytopenia, agranulocytosis (incidence unknown *), thrombocytopenia (0.1-5%)

5. Interstitial pneumonia (less than 0.1%)

6. Liver dysfunction (less than 0.1-5%), jaundice (incidence unknown)

7. Acute renal failure (less than 0.1%)

Other side effects <0.1% or more or side effects of unknown frequency>

1. Hypersensitivity: rash, rash, pruritus, photosensitivity, erythema

2. Cardiovascular: Palpitations, tachycardia, burning, elevated blood pressure, decreased blood pressure, arrhythmia such as atrial fibrillation, supraventricular tachycardia, supraventricular extrasystole, ventricular extrasystole, etc.

3. Psycho-nervous system: headache / heavy headache, dizziness, insomnia, numbness

4. Gastrointestinal: abdominal pain, nausea / vomiting, loss of appetite, diarrhea, heartburn, bloating, abnormal taste

5. Blood: Anemia, leukopenia

6. Bleeding tendency: Subcutaneous bleeding, hematuria, etc.

7. Liver: Increase in AST (GOT), ALT (GPT), Al-P, LDH, etc.

8. Kidney: BUN increased, creatinine increased, uric acid level increased, pollakiuria

9. Others: sweating, edema, chest pain, elevated blood sugar, tinnitus, malaise, conjunctivitis, fever, hair loss

Aspirin

Serious side effects

1. Shock, anaphylaxis-like symptoms (incidence unknown)

2. Bleeding (incidence unknown) <Intracranial hemorrhage such as cerebral hemorrhage, pulmonary hemorrhage, gastrointestinal hemorrhage, epistaxis, fundus hemorrhage, etc.>

3. Mucocutaneous ocular syndrome (Stevens-Johnson syndrome), toxic epidermal necrolysis (Lyell syndrome), exfoliative dermatitis (incidence unknown)

4. * Aplastic anemia, thrombocytopenia, leukopenia (incidence unknown)

5. Asthma attack (incidence unknown)

6. Liver dysfunction, jaundice (incidence unknown)

7. Stomach ulcer, small intestine / large intestine ulcer (incidence unknown)

Other side effects <0.1% or more or side effects of unknown frequency>

Gastrointestinal disorders (gastrointestinal disorders, vomiting, abdominal pain, heartburn, constipation, diarrhea, esophagitis, lip swelling, hematemesis, nausea, nausea, loss of appetite, gastric discomfort)

Hypersensitivity (urticaria, rash, edema)

Skin (itch, rash, wheal, sweating)

Psycho-nervous system (dizziness, agitation, headache)

Liver (AST (GOT) elevation, ALT (GPT) elevation)

Cardiovascular (lowering blood pressure, vasculitis, epigastric pain)

Respiratory (bronchitis, epistaxis, rhinitis)

Sensory organs (tinnitus, deafness)

Others (hyperventilation, metabolic acidosis, malaise)

Clopidogrel

Serious side effects

1. Bleeding (intracranial hemorrhage, gastrointestinal hemorrhage, etc.) [Intracranial hemorrhage such as cerebral hemorrhage (less than 1%), subdural hematoma (less than 0.1%), etc.] [Vomiting (incidence unknown)), melena, gastrointestinal hemorrhage, Bleeding of the fundus of the eye (less than 1%), joint hematoma (less than 0.1%), etc.]

2. Gastric / duodenal ulcer (incidence unknown))

3. Liver dysfunction, jaundice

4. Thrombotic thrombocytopenic purpura (TTP) (incidence unknown))

5. Interstitial pneumonia (less than 0.1%)

6. Pancytopenia including thrombocytopenia, agranulocytosis, and aplastic anemia (incidence unknown))

7. Toxic epidermal necrolysis (TEN), mucocutaneous ocular syndrome (Stevens-Johnson syndrome), erythema multiforme (incidence unknown))

8. Rhabdomyolysis (incidence unknown))

Other side effects <0.1% or more or side effects of unknown frequency>

Blood (subcutaneous bleeding, anemia, purpura (disease), nasal bleeding, prolongation of hemostasis, eye bleeding, gingival bleeding, hemorrhage bleeding, bloody sputum, puncture site bleeding, post-treatment bleeding, hemoglobin depletion, erythrocyte depletion, hematocrit depletion, leukocyte depletion, favorable (Medium hypoplasia, eosinophilia, serum sickness)

Liver (Al-P elevation, LDH elevation, serum bilirubin elevation)

Digestive system (digestive discomfort, gastroenteritis, stomatitis, abdominal pain, vomiting, diarrhea, loss of appetite, constipation, esophagitis, vomiting, colitis (ulcerative colitis, lymphocytic colitis), pancreatitis)

Metabolic disorders (increased triglyceride, increased CK (CPK), increased total cholesterol, decreased total protein, increased K, decreased albumin)

Hypersensitivity (rash, itching, eczema, urticaria, erythema, anaphylactic reaction, patchy papular rash, angioedema, bronchospasm)

Skin (blister rash, lichen planus)

Psychoneurological system (headache, hypertension, dizziness, numbness, musculoskeletal stiffness (stiff shoulders, finger stiffness), impaired consciousness, insomnia, loss of consciousness, voice modulation, hypotension, epilepsy, drowsiness, cutaneous hyperesthesia, tearing, mood swings )

Cardiovascular (edema, tachycardia, arrhythmia, vasculitis)

Kidney (elevated BUN, elevated blood creatinine, increased urine protein, hematuria, abnormal urinary sediment, positive urine sugar, renal dysfunction, glomerulopathy)

Others (hot flashes, arthritis, fever, abnormal feeling (floating feeling, feeling unwell), myalgia, arthralgia)

Prasugrel

Serious side effects

1. Bleeding (intracranial hemorrhage, gastrointestinal hemorrhage, etc.) [Intracranial hemorrhage such as cerebral hemorrhage (less than 1%), subdural hematoma (less than 0.1%), etc.] [Vomiting (incidence unknown)), melena, gastrointestinal hemorrhage, Bleeding of the fundus of the eye (less than 1%), joint hematoma (less than 0.1%), etc.]

2. Gastric / duodenal ulcer (incidence unknown))

3. Liver dysfunction, jaundice

4. Thrombotic thrombocytopenic purpura (TTP) (incidence unknown))

5. Interstitial pneumonia (less than 0.1%)

6. Pancytopenia including thrombocytopenia, agranulocytosis, and aplastic anemia (incidence unknown))

7. Toxic epidermal necrolysis (TEN), mucocutaneous ocular syndrome (Stevens-Johnson syndrome), erythema multiforme (incidence unknown))

8. Rhabdomyolysis (incidence unknown))

Other side effects <0.1% or more or side effects of unknown frequency>

Blood (subcutaneous bleeding, anemia, purpura (disease), nasal bleeding, prolongation of hemostasis, eye bleeding, gingival bleeding, hemorrhage bleeding, bloody sputum, puncture site bleeding, post-treatment bleeding, hemoglobin depletion, erythrocyte depletion, hematocrit depletion, leukocyte depletion, favorable (Medium hypoplasia, eosinophilia, serum sickness)

Liver (Al-P elevation, LDH elevation, serum bilirubin elevation)

Digestive system (digestive discomfort, gastroenteritis, stomatitis, abdominal pain, vomiting, diarrhea, loss of appetite, constipation, esophagitis, vomiting, colitis (ulcerative colitis, lymphocytic colitis), pancreatitis)

Metabolic disorders (increased triglyceride, increased CK (CPK), increased total cholesterol, decreased total protein, increased K, decreased albumin)

Hypersensitivity (rash, itching, eczema, urticaria, erythema, anaphylactic reaction, patchy papular rash, angioedema, bronchospasm)

Skin (blister rash, lichen planus)

Psychoneurological system (headache, hypertension, dizziness, numbness, musculoskeletal stiffness (stiff shoulders, finger stiffness), impaired consciousness, insomnia, loss of consciousness, voice modulation, hypotension, epilepsy, drowsiness, cutaneous hyperesthesia, tearing, mood swings )

Cardiovascular (edema, tachycardia, arrhythmia, vasculitis)

Kidney (elevated BUN, elevated blood creatinine, increased urine protein, hematuria, abnormal urinary sediment, positive urine sugar, renal dysfunction, glomerulopathy)

Others (hot flashes, arthritis, fever, abnormal feeling (floating feeling, feeling unwell), myalgia, arthralgia)

Appendix 7. Anonymization number comparison table

How to use the anonymization number comparison table

In clinical research, from the viewpoint of privacy protection, no personally identifiable information can be included in the case report form. All are managed by registration number.

Therefore, it is necessary for the facility to manage the correspondence between the individual patient and the registration number.

The subject identification code is the only information that can be used to identify a patient without disclosing personal information.

* 1: The subject identification code does not include personal information, and please attach an arbitrary code that is unique.

<Not good example>

Yamataro ・ ・ ・ ・ ・ ・ ・ ・ ・ ・ ・ ・ ・ It can be guessed as Yama ○ Taro.

YT19370304 ・ ・ ・ ・ ・ It can be inferred that the initial YT was born on March 4, 1945.

Ya 200512345 ・ ・ ・ ・ ・ ・ ・ ・ ・ ・ ・ ・ ・ The medical record number cannot be used because the individual can be easily identified.

0783031917 ・ ・ ・ ・ ・ ・ Personal information such as the patient's phone number cannot be used.

When creating a case report, be sure to check the correspondence between the patient's chart and the registration number on this anonymized number comparison table.

This table may be created for each teacher or managed for each facility.
